# Sanitizer-associated systemic side effects in the era of COVID-19: a pharmacovigilance study

**DOI:** 10.1186/s43088-022-00263-7

**Published:** 2022-06-13

**Authors:** Hasnaa Osama, Mona A. Abdelrahman

**Affiliations:** Department of Clinical Pharmacy, Faculty of Pharmacy, Beni-Suef University, Beni Suef, Egypt

**Keywords:** Disinfectant, Hand sanitizer, Toxicity, COVID-19, Pharmacovigilance

## Abstract

**Background:**

The practice of proper hand sanitization became of utmost importance and one of the best protective measures during the pandemic outbreak of COVID-19. However, misuse of disinfectants can be an overwhelming issue because of increasing demands, public panic, and unawareness, which can negatively affect human health and the environment. Therefore, we aimed to determine whether the outbreak was associated with increased reports of adverse events related to hand sanitizers through the data of adverse events reported to the pharmacovigilance database of the Food and Drug Administration Adverse Event Reporting System (FAERS). FAERS database was analyzed for hand sanitizer reports including alcohol-based and alcohol-free formulations.

**Results:**

Adverse events reports associated with hand sanitizers increased significantly by 2020 with variable severity degrees, noting that most serious cases were reported with alcohol-based sanitizers.

**Conclusion:**

Based on data mining of the FAERS database, we claim that the increased reports associated with alcohol hand sanitizer use deserve attention. However, FAER’s database has some limitations, such as case duplication and lack of a control group. Hence, further monitoring with more robust sources of data sources is critically needed.

## Background

Hands might be polluted easily from droplet transmission when direct contact with sneezes and coughs [1]. Regular Hand hygiene is one of the most important steps of infection prevention measures during the COVID-19 pandemic outbreak [2]. It is fundamental to interrupt the virus transmission chain with suitable hand sanitization [1]. There are various hand hygiene products existing; however, their safety and efficacy fluctuate [3]. Alcohol-based hand sanitizers, as the chief constituent, are highlighted as the best hand hygiene practices [1]. Antiviral activity of alcohol is attributed to its penetrating ability to the viral membrane, denaturing and coagulating its proteins, interfering with its cellular metabolism, and finally analyzing viral components [4, 5]. On the other hand, the American Association of Poison Control Center informed 9504 cases of children less than 12 years exposed to alcoholic hand sanitizer. The center presented that even a minor quantity of alcohol can cause alcohol poisoning in children, which is apparent with vomiting, drowsiness, and confusion, and in severe cases, respiratory arrest and death [6]. It also causes dry skin through skin absorption, and it is possible to cause skin cancer, and carcinogenicity is still unclear due to a lack of up-to-date research [6, 7]. In addition, applying ethanol to immature skin can lead to reactions and systematic toxicity [7, 8], which is exhibited in ethanol intoxication due to percutaneous absorption, especially in children below 33 months of age [6]. Unlike alcoholic-based hand sanitizers, preparations without alcohol such as formulations based on benzalkonium chloride are commonly less effective [3]. Moreover, recent evidence proposes it is irritating with the potential to cause contact dermatitis more frequently than previously assumed [6, 9].

Hence, this study aims to identify and analyze the number of adverse events reported as a consequence of hand sanitizer use in the pharmacovigilance database available to the public.

## Methods

The publicly available FDA's Adverse Event Reporting System (FAERS) reports were analyzed for the period between January 2010 and March 2022. This database is accessible via a querying tool on an online dashboard and downloadable files [10]. The dashboard contains files of voluntary adverse drug reaction (ADR) reports. These reports were sent to the FDA either directly (e.g., healthcare specialists or consumers) or indirectly through mandatory drug manufacturer reports from different countries. The event report, also known as an individual case safety report (ICSR), contains data classified as identification document (ID) of the case, suspected product name and active ingredients, indication for use, outcome (serious/non-serious), country, reporter type, and event date. These adverse event reports also include demographics such as gender (male, female, or unknown) and age of the case.

### Search strategy and data analysis

The included reports in the FAERS database are organized based on the Medical Dictionary for Regulatory Activities (MedDRA) classification system. Reports of side effects associated with hand sanitizers used for prophylaxis against COVID-19 during the period from 2019 up to 2021 were stratified and analyzed. Also, reports starting from 2013 were included. Data duplicates were identified using case numbers and merged as one case. Duplication of data could be attributed to reporting by multiple caregivers of one patient. In addition, reports submission from pharmaceutical companies is mandatory once ADR is identified [11]. The total number of reports for hand sanitizers with or without alcohol was calculated. Hand sanitizers without alcohol or containing other constituents such as benzalkonium chloride were considered as the comparator group. For each year, the proportional reporting ratios (PRRs) were estimated as ([number of serious adverse events for alcohol-based sanitizers]/[Total number of reports for alcohol sanitizers])/([Number of serious adverse events for non-alcohol sanitizers]/[total number of reports for non-alcohol sanitizers]) [12]. For quality assurance of data collection and analysis, the pharmacovigilance FAERS database was independently assessed by two reviewers.

## Results

**Fig. 1:**
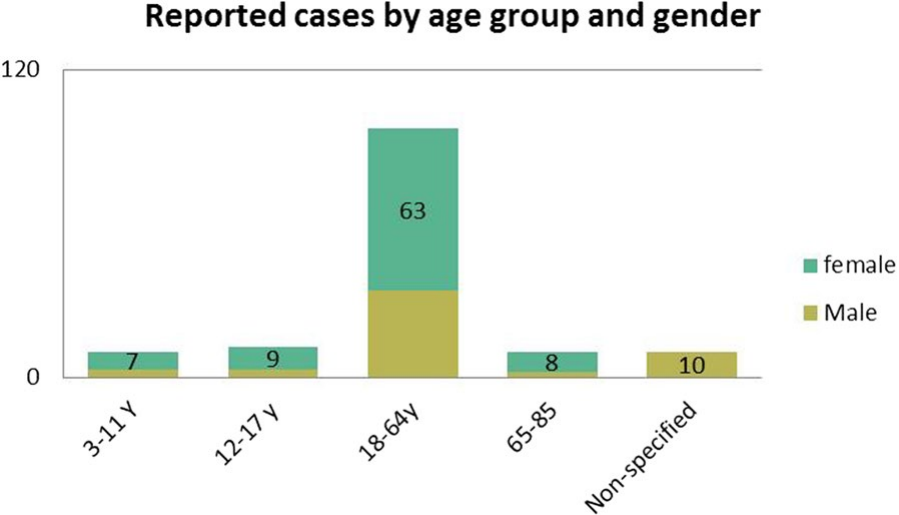
Reported cases of adverse events associated with sanitizers use by age group and gender

A preliminary query using FDA adverse drug events dashboard revealed that about 139 cases reported adverse effects following hand sanitizers used for infection prophylaxis and most cases were with alcohol-based active ingredients. Only a few cases were reported before 2015. The majority of cases were reported in 2020, increasing from 4 cases (2.87%) by 2019 to over 120 cases (86.33%) by June 2021, where sanitizers were used for infection prophylaxis during the COVID-19 pandemic. Demographic and clinical data were reported for most cases (92.8%), with only a limited number of cases (7.19% of the total reported cases) were stated as non-specified (Table 1). Most reported adverse events were among adults with a mean age (SD) of 44.8 (16.95) years, with 16 children aged between 3 and 17 years of age (Fig. 1). As of March 2020, relative to the comparator group (non-alcoholic sanitizers), the estimated signal score using PRR for alcohol-based sanitizers was 2.14 compared to only 0.035 before the aforementioned year.

**Table 1:** Characteristics of adverse reactions which reported by food and drug administration adverse event reporting system dashboard

Suspect active ingredients	All adverse reactions *N* (%)	Reported adverse reactions *N* (%)		Gender *N* (%)			Age mean (SD)
		Serious	Non-serious	Male	Female	Not specified	
Alcohol	114 (82.01)	64 (56.41)	50 (43.85)	37 (32.46)	70 (61.4)	7 (6.14)	52.6 (18.7)
Benzalkonium Chloride; Alcohol	10 (7.19)	8 (80)	2 (20)	2 (20)	7 (70)	1 (10)	34.7 (10.9)
Benzalkonium Chloride	15 (10.79)	9 (60)	6 (40)	3 (20)	10 (66.7)	2 (13.3)	44.3 (9.2)

**Fig. 2:**
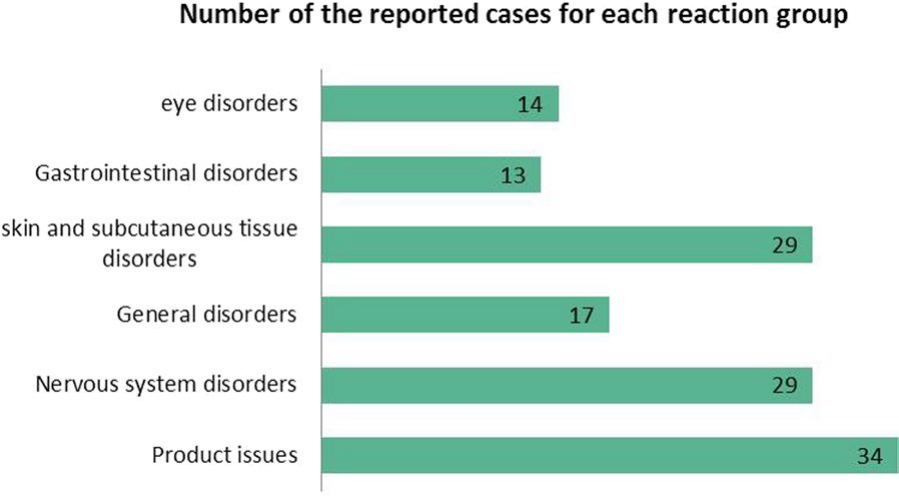
Number of the reported cases of each reaction group

The severity of reactions associated with hand sanitizers was classified as serious and non-serious by the FAERS database; where serious cases accounted for 58.27%, most of them reported with alcohol-based sanitizers, with variability from nausea, headache, gastrointestinal disorders, rashes, and skin reactions to death in few cases (Fig. 2), while non-serious cases accounted for 41.73%, where alcohol-based sanitizers were associated with most cases (about 50 reports) as well.

## Discussion

During the era of public health emergency due to COVID-19 outbreak, several organizations provided extensive and critical information on hand and surface disinfectants to protect the health of individuals. During this endeavor, hand sanitizers have gained lots of attention as a prophylactic agent against COVID-19. In the same line, we performed the first study of the possible adverse reactions associated with sanitizers by using the FAERS pharmacovigilance database and an in-depth analysis of individual case reports.

The widespread use of disinfectants across the globe can result in secondary overwhelming of human health and the ecosystem. Recent studies have reported that the frequent use of disinfectant compounds, including alcohol-based and alcohol-free formulations, were linked to a considerable risk of respiratory diseases and eye irritation on both health workers and regular users of sanitizers [4–6]. In our study, the retrieved and analyzed data from the FAERS database showed a significant association with alcohol-based sanitizers than alcohol-free formulations.

Benzalkonium chloride, which is known as quaternary ammonium compound, is considered as the principal component of alcohol-free disinfectants with low potency and less efficacy as compared to alcohol-based preparations [13–15]. Also, it came in second place in terms of the number of reported adverse effect cases with a considerable number of serious adverse effects.

Several published studies reported the risk of inhalation of chemical residues during the application of alcohol-based sanitizer to the skin with considerable risk of spoiling the quality of air indoors resulting in potential hazards such as respiratory disorders, nervous system disorders allergic skin reactions, and eye irritation [9, 13, 16]. Along with headache, dizziness, and nausea which were commonly reported, herein, we also observed novel adverse effects including migraine (*n* = 5) and memory impairment (*n* = 1). Serious life-threatening adverse outcomes were reported in a few cases (*n* = 8).

Therefore, The FDA agency recommended against the overuse of hand sanitizers and the application of sanitizers in an area with well ventilation [17].

There are several limitations in this retrospective study. Using the FAERS database can result in many instances of duplication errors since the same report can be submitted by the consumer and the manufacturer. Additionally, necessary information and adverse outcomes may not be reported in some cases. Furthermore, the publicity and the widespread product use can affect the number of reports with a potential risk of bias. The continuous updates of information in the FAERS database result in variability in the number of individual cases, which may increase or decrease over time.

## Conclusion

Given the widespread use of hand sanitizers, the remarkable increase in the adverse events related to sanitizer use reported to the FAERS database, and the anticipated continuous use in the future as a prophylactic measure during the era of the pandemic, this study highlights the need for cautious use of disinfectants. Moreover, further monitoring using adjusted methods with less bias is warranted.

## Data Availability

The datasets used and/or analyzed during the current study are available from the corresponding author on reasonable request.

## Abbreviations

ADR: Adverse drug reaction
COVID-19: Coronavirus disease of 2019
FDA: Food and Drug Administration
FAERS: Food and Drug Administration Adverse Event Reporting System
ID: Identification document
ICSR: Individual case safety report
MedDRA: The Medical Dictionary for Regulatory Activities
PRR: Proportional reporting ratios
